# Honey bees increase social distancing when facing the ectoparasite *Varroa destructor*

**DOI:** 10.1126/sciadv.abj1398

**Published:** 2021-10-29

**Authors:** Michelina Pusceddu, Alessandro Cini, Simona Alberti, Emanuele Salaris, Panagiotis Theodorou, Ignazio Floris, Alberto Satta

**Affiliations:** 1 Department of Agricultural Sciences, University of Sassari, viale Italia 39A, 07100 Sassari, Italy.; 2 Centre for Biodiversity and Environment Research, University College London, Gower Street, London WC1E 6BT, UK.; 3 Department of Life Sciences and Systems Biology, University of Turin, via Accademia Albertina 13, 10123 Turin, Italy.; 4 General Zoology, Institute for Biology, Martin Luther University Halle-Wittenberg, Hoher Weg 8, 06120 Halle (Saale), Germany.

## Abstract

Social distancing in response to infectious diseases is a strategy exhibited by human and nonhuman animals to counteract the spread of pathogens and/or parasites. Honey bee (*Apis mellifera*) colonies are ideal models to study this behavior because of the compartmentalized structure of these societies, evolved under exposure to parasite pressure and the need to ensure efficient functioning. Here, by using a combination of spatial and behavioral approaches, we investigated whether the presence of the ectoparasite mite *Varroa destructor* induces changes in the social organization of *A. mellifera* colonies that could reduce the spread of the parasite. Our results demonstrated that honey bees react to the intrusion of *V. destructor* by modifying space use and social interactions to increase the social distancing between young (nurses) and old (foragers) cohorts of bees. These findings strongly suggest a behavioral strategy not previously reported in honey bees to limit the intracolony parasite transmission.

## Teaser

Honey bees modify their social organization to hinder the spread of parasites within the colony.

## INTRODUCTION

Social insects are particularly vulnerable to pathogens and parasites owing to the dense network of contacts among highly related nestmates and the large amounts of food stored in a nest under relatively stable environmental conditions (*1*). To counteract disease pressure, social insects have evolved, in addition to individual immune responses, many forms of social immunity, i.e., strategies based on the cooperation of the individual group members (*2*). The latter occur at the behavioral, physiological, and organizational level and can act synergistically to avoid invasion, establishment, and replication of pathogens or parasites inside the colony (*2*, *3*).

An aspect of particular importance is colony organization, i.e., the pattern of social interactions among colony members across space and time. While ergonomic optimization selects dense and interconnected societies (*4*), pathogen and/or parasite pressure is a crucial factor driving insect colony organization (*1*) that favors mechanisms that limit interactions between individuals to reduce the risk of disease spread (*5*–*7*). Therefore, it can be predicted that the rate of contacts between nestmates would be limited by spatial and behavioral compartmentalization of the different cohorts of individuals, according to their age, role (caste), and activity (task) (*6*–*8*). Such a “constitutive organizational immunity” takes place also in the absence of a disease challenge, thus acting as a prophylactic immune defense (*2*, *6*, *8*). A clear-cut demonstration of this theoretical model in social insects was given by Stroeymeyt *et al.* (*9*).

Being threatened by dozens of pests and diseases (*10*), honey bee colonies might benefit from constitutive organizational immunity. High-resolution spatial and social network analyses demonstrated that honey bee colonies are organized into two main compartments: the outer one occupied by the foragers (old bees) clumped together near the entrance of the hive, and the innermost compartment inhabited by nurses (young bees) with the queen, which spend most of their time on brood cells (*11*). This within-colony spatial segregation leads to a lower frequency of interactions between the two compartments than within each compartment and allows the most valuable individuals, i.e., queen, young bees, and brood, to be protected from the outside environment and thus from the arrival of diseases (*5*, *11*).

This constitutive type of immune defense could be enriched by reactive strategies, i.e., the modification of the colony social networks and space use inside the nest when challenged by a parasite or a pathogen (induced organizational immunity), with the aim of further reducing the risk of spreading a disease (*8*, *12*). This hypothesis has been recently confirmed in the ant *Lasius niger* infected with the fungal pathogen *Metarhizium brunneum*, which induced behavioral changes not only among pathogen-exposed foragers but also among their nestmates, thus reducing individual contamination risk (*9*). Similarly, honey bee colonies challenged with an experimental inoculation of Israeli acute paralysis virus reduced social contacts between the colony members, suggesting an adaptive social immune response by hosts to reduce pathogen transmission (*13*).

These studies demonstrated induced organizational immunity in response to microbial diseases, but we still lack evidence for such a reactive social immune response when social insect colonies are challenged by arthropod ectoparasites, such as mites. Nests of social insects harbor a wide variety of mites (*14*), some of which can have a substantial impact on colony fitness (*15*–*19*). *Varroa destructor* is among the most serious threats to honey bees worldwide (*17*, *19*) and has played a fundamental role in the decline of honey bee colonies all over the Northern Hemisphere in the past decades (*20*, *21*). This mite causes a number of detrimental effects on bees at the individual and colony level (*22*, *23*), including the transmission of bee viruses (*24*). The intrusion of the parasite inside uninfested colonies normally occurs through foragers of the same colony, which may encounter the parasite on flowers (*25*), but also via foragers from foreign colonies because of drifting or robbing phenomena (*26*). To reproduce, the foundress mite needs to enter a brood cell with a mature bee larva (*17*, *19*). At the end of the honey bee pupal stage, the mother mite and its progeny will come out from the cell with the emerging bee (*17*). Then, the dispersal of the parasite within the hive takes place by parasitizing mainly nurse bees (phoretic phase) (*27*).

In this work, we investigated whether the presence of the ectoparasite mite *V. destructor* in honey bee colonies induces changes in the social immunity strategies that could reduce the spread of the parasite. We focused on space use and social interaction patterns, which are two features affecting organizational immunity (*11*). Our study was conducted at two levels: observation of whole colonies undergoing natural *V. destructor* infestations in field settings to observe broad-scale changes in specific immune defense strategies, and high-resolution observation of individual behavior in small groups of caged bees experimentally infested with *V. destructor* in the laboratory, to assess the fine-scale changes in social behavior due to *Varroa* infestation. These two levels allowed us to test specific predictions deriving from the social immunity theory (*2*).

In the whole-colony experiment, we made comparative behavioral observations on *Varroa*-infested and *Varroa*-free colonies, placed in observation hives. The different level of infestation in the two experimental groups was obtained by applying different acaricidal treatments to randomly selected hives. We monitored two types of behavior strongly involved in the dispersion of the parasite (*5*): foraging dances and allogrooming. Once inside the hive, foragers can perform foraging (round and waggle) dances to communicate the exact position of a food source to other foragers (dance followers) (*28*). Foragers prefer to dance on the combs near the center of the hive congregated in areas of the lower half that are nearest to the entrance (*29*). Because foragers represent a relevant entry route of *V. destructor* into the colony, it would be advantageous to limit the social contacts between foragers and other colony members when the mite is present. Therefore, a prediction would be that, under parasite pressure, foragers would change their space use, by spatially shifting their dances toward the periphery of the colony (prediction 1).

Allogrooming is a social behavior by which a bee removes foreign particles and parasites from another bee (*30*, *31*). *Apis mellifera* is able to remove and kill *Varroa* through allogrooming activity, albeit less effectively than *Apis cerana* (*32*). Generally, this sporadic behavior is exhibited by young bees, at an age between 6 and 11 days (*33*), when they mainly act as nurse bees (*34*). Under a *V. destructor* infestation, it would likely be better for the bees to concentrate the allogrooming effort in the core of the colony, i.e., in the region of the comb with brood, where nurses remain and where newly emerged bees carrying mites might emerge more frequently (prediction 2). Therefore, we made an overall prediction of an opposite pattern between foraging dances and allogrooming in infested colonies, with centrifugal and centripetal shifts in their occurrence: We expected foraging dances to prevail in the periphery and allogrooming to be concentrated in the core of the colony.

In the laboratory experiment, the high-resolution observations of individual behavior in experimentally infested bee groups and *Varroa*-free bees were conducted on young bee cohorts (1-day-old bees), as they represent the vehicle by which post-pioneer mite generations emerge from the cells and spread in the nest (*17*, *19*). We analyzed whether the presence of *Varroa* influenced individual social behavior and how this affected the small-scale social network cohesion, by focusing on three common social behaviors that are also involved in the fight with and spread of the mite: allogrooming, antennation, and trophallaxis. Antennation is the main method used by honey bees for nestmate recognition (*35*). Trophallaxis is a process in which a bee distributes liquid food to other bees, with 1-day-old workers being more frequently donor bees (*36*). We predicted to find different patterns of individual social behavior under parasite pressure (prediction 3), in accordance with the social immunity theory (*2*). More specifically, we predicted an increase in allogrooming and a decrease in antennation and trophallaxis, because the former normally limits the dispersion of the disease, whereas the latter two normally increase it. The balance between an increase in allogrooming and a decrease in antennation and/or trophallaxis would affect the cohesion of the social network (i.e., how well the group is connected) in infested groups (prediction 4). As allogrooming is a sporadic social interaction, especially if compared to antennation and trophallaxis (*33*), we predicted that the effect of reduced antennation and trophallaxis would outweigh the expected increase in allogrooming. Therefore, a decrease in network connectivity and node centrality (the extent to which a bee is well connected within the network of social interactions, considering both performed and received interactions, i.e., outgoing and incoming centrality, respectively) in infested groups at the whole network level and/or a decrease in individual centrality in infested groups (especially in infested individuals) at the single node (bee) level would be expected.

Our study demonstrates that honey bee colonies react to the invasion of an ectoparasitic mite with significant changes in behavioral traits associated with social immunity (space use and social interactions) at both the whole colony and the individual level. These findings strongly suggest that honey bees limit the spread of parasites within the colony by social distancing.

## RESULTS

### Whole-colony experiment

#### Prediction 1

**Fig. 1.**
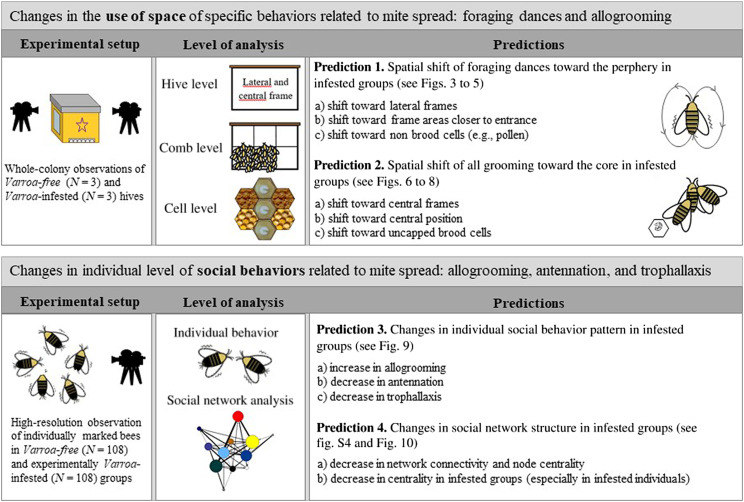
Summary of experimental design. Does infestation by *Varroa* mites induce changes in use of space and social behavior in the honey bee?

To test prediction 1 (Fig. 1), i.e., spatial shift in foraging dances toward the periphery of the colony under parasite pressure, we verified whether foraging dances occurred mostly on lateral frames rather than on central ones (prediction 1a) and/or closer to the entrance of the hive rather than on the central part of the comb (prediction 1b), in *Varroa*-infested hives compared with *Varroa*-free hives. Moreover, as the colonization of brood cells is a crucial step in *Varroa* reproduction, a further prediction that we verified was a decrease in foraging dances on brood cells in infested colonies compared to noninfested colonies (prediction 1c). To determine more easily the position in the comb in relation to the hive entrance where the behavior took place, the observed frame was divided into six portions of equal area as shown in fig. S1.

The average colony strength was similar in the two groups (number of adult bees and sealed brood cells, *Varroa*-free: 22,581 ± 1761 versus *Varroa*-infested: 21,793 ± 3168; mean + SE; Mann-Whitney *U* test; *U* = 7, df = 4, *P* > 0.05; table S1), whereas the infestation level (mean percentage of mites per adult bees ± SE) in the *Varroa*-infested group was significantly higher compared to the *Varroa*-free group (6.18 ± 0.34% versus 0.11 ± 0.11%, Mann-Whitney *U* test; *U* = 10, df = 4, *P* < 0.05; table S1). *Varroa*-free hives were obtained by treating half of the colonies with trickled oxalic acid every week, for three consecutive weeks, starting from 2 months before the start of the observations. In the *Varroa*-infested group, only the first treatment with trickled oxalic acid was applied, 2 months before the beginning of the observations, after which *Varroa* infestation grew naturally.

By checking videos recorded inside the hives of the two experimental groups, we detected in the same time interval (13 hours and 30 min per group) a total of 394 and 453 events of foraging dances (round and waggle) in *Varroa*-free and in *Varroa*-infested colonies, respectively. The frequency of dances did not differ between the two experimental groups [generalized linear mixed model (GLMM) using day, time slot of recording, and hive code as random factors; χ^2^ = 0.254, df = 1, *P* = 0.613; fig. S2A] also when considering the two types of dance (round and waggle) (interaction effect between experimental group and type of dance; GLMM using day, time slot of recording, and hive code as random factors; χ^2^ = 2.098, df = 1, *P* = 0.147; fig. S2B).

**Fig. 2.**
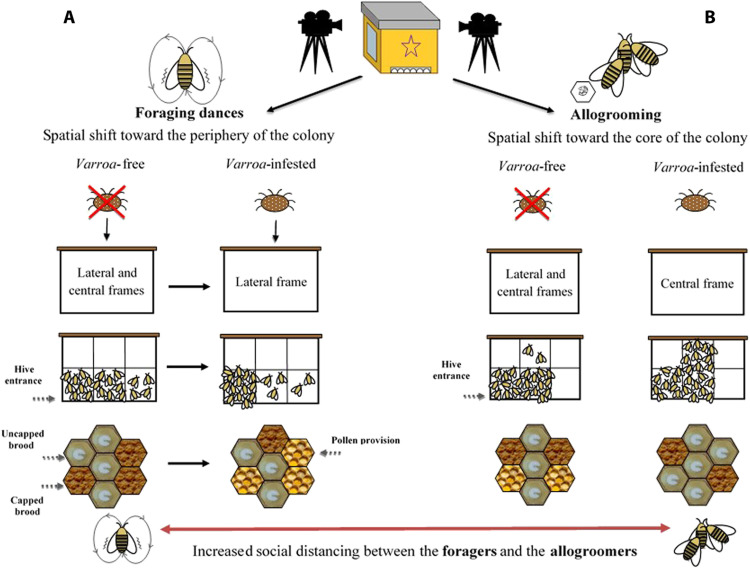
Induced organizational immunity. Spatial shift in foraging dances (**A**) and allogrooming behavior (**B**) observed in the whole-colony experiment.

**Fig. 3.**
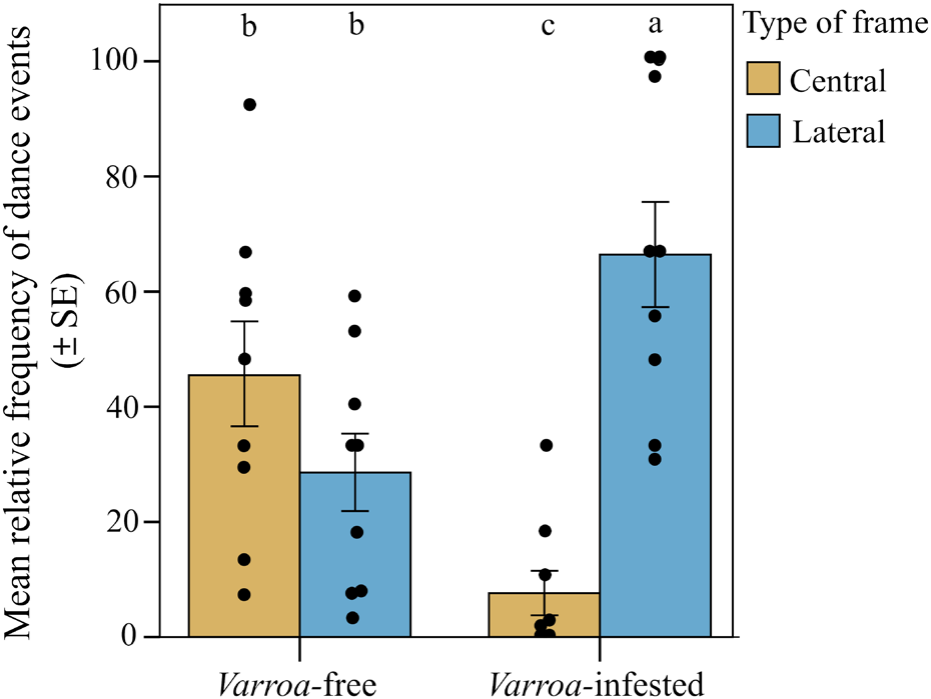
Spatial shift in foraging dances. Relative frequency (mean ± SE) of dance (round and waggle) events per hive and day of observation detected in *Varroa*-free and *Varroa-*infested colonies depending on the type of frame (central or lateral). Bars with different letters are significantly different (*P* < 0.05).

**Fig. 4.**
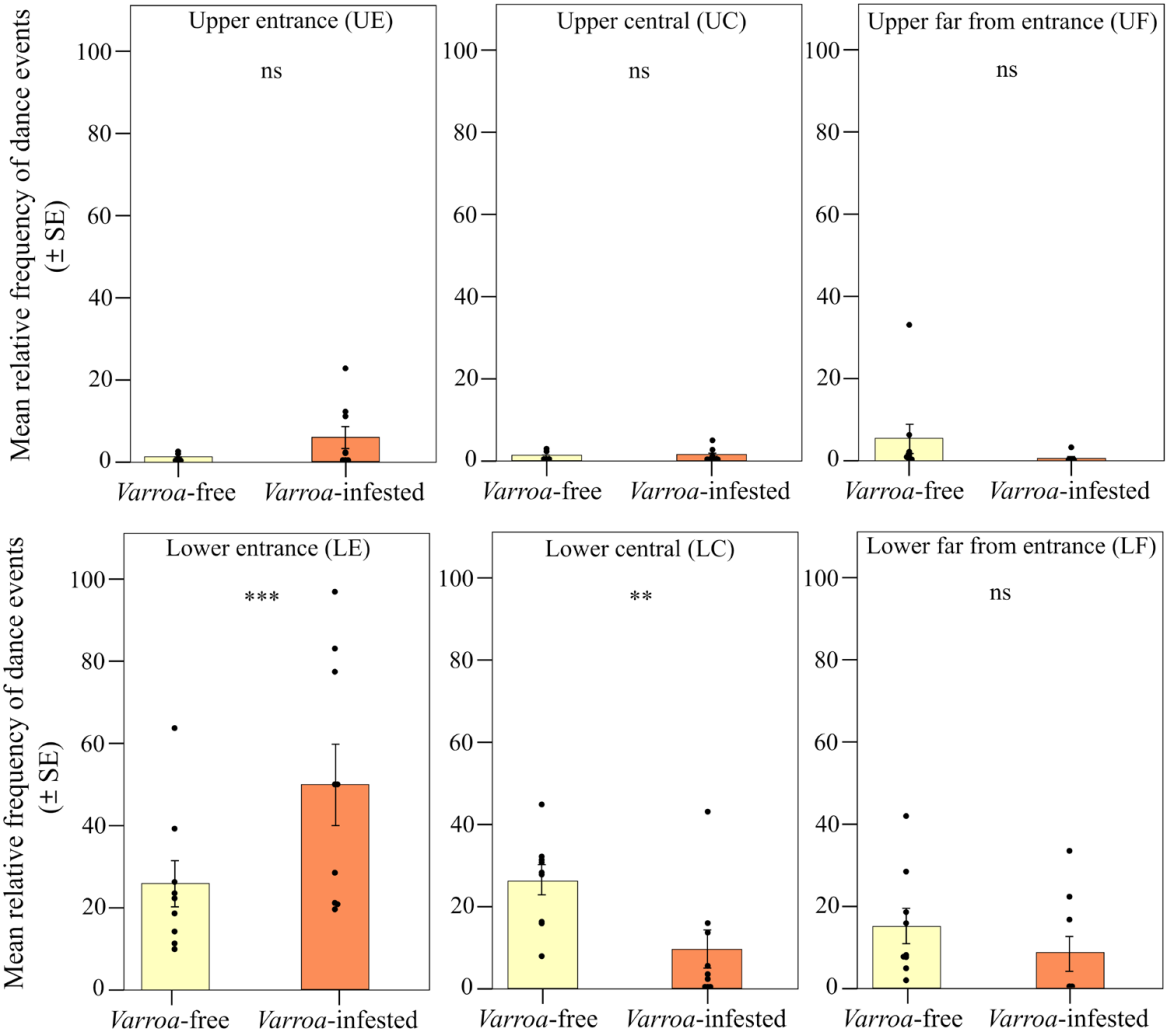
Spatial shift in foraging dances. Relative frequency (mean ± SE) of dance (round and waggle) events per hive and day of observation detected in *Varroa*-free and *Varroa-*infested colonies depending on the position in the frame in relation to the hive entrance [lower and upper position near the entrance (LE and UE), lower and upper central position (LC and UC), and lower and upper position far from the entrance (LF and UF)]. Not significant (ns), *P* > 0.05; **P < 0.01; and ***P < 0.001.

**Fig. 5.**
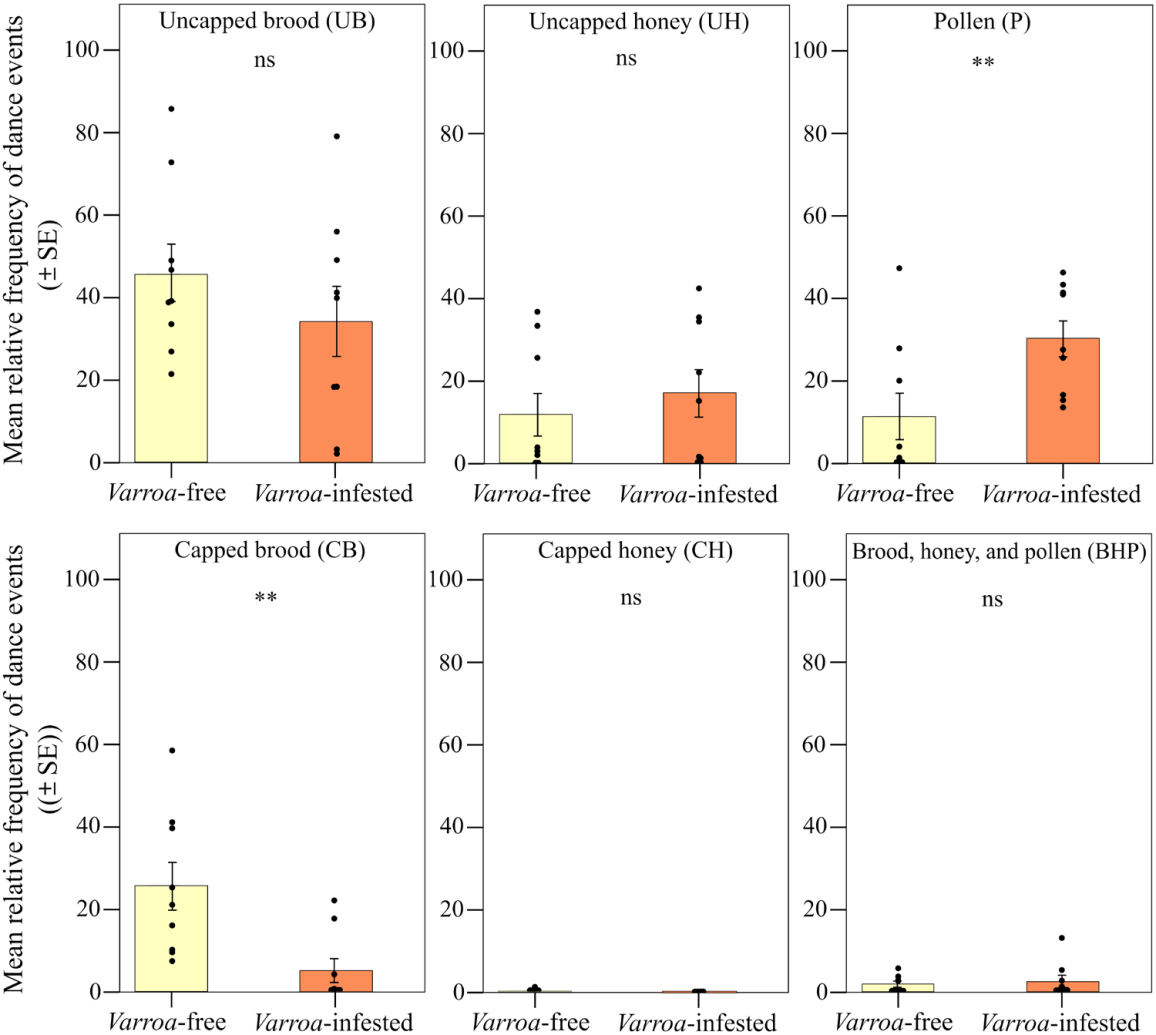
Spatial shift in foraging dances. Relative frequency (mean ± SE) of dance (round and waggle) events per hive and day of observation detected in *Varroa*-free and *Varroa-*infested colonies depending on the type of substrate [uncapped and capped brood (UB and CB); uncapped and capped honey (UH and CH); pollen (P); and a mix between brood, honey, and pollen (BHP)]. ns, *P* > 0.05; and **P < 0.01.

We found strong support for the prediction of a peripherical shift in the expression of foraging dances (Fig. 2A). When comparing the two types of frames (central and lateral), we found significant differences in the relative frequency of foraging dances (round and waggle) within the *Varroa*-infested colonies and between *Varroa*-infested and *Varroa*-free colonies [interaction effect between experimental group and frame position; linear mixed model (LMM) using day, time slot of recording, and hive code as random factors; χ^2^ = 25.227, df = 1, *P* < 0.001]. In particular, confirming prediction 1a, the relative frequency of dances was significantly higher in the lateral frame and significantly lower in the central frame in the infested group compared to the uninfested group [LMM using day, time slot of recording, and hive code as random factors, Tukey post hoc test with false discovery rate (FDR) correction, central frame; *z* = 3.552, *P* < 0.001; lateral frame; *z* = 3.550, *P* < 0.001; Fig. 3]. In addition, considering the two experimental groups independently, we found a higher relative frequency of foraging dances in the lateral food frame than in the central brood frame in the infested group (LMM using day, time slot of recording, and hive code as random factors, Tukey post hoc test with FDR correction; *z* = 5.519, *P* < 0.001; Fig. 3), whereas no significant differences were observed between these two types of frame in *Varroa*-free colonies (LMM using day, time slot of recording, and hive code as random factors, Tukey post hoc test with FDR correction; *z* = 1.584, *P* = 0.113; Fig. 3). Foraging dance behavior also varied depending on the position within the comb in relation to the hive entrance (frame level; fig. S1), confirming prediction 1b (interaction effect between experimental group and comb position; LMM using day, time slot of recording, and hive code as random factors; χ^2^ = 29.941, df = 5, *P* < 0.001; Fig. 4). Dances were more frequent in the lower position near the entrance (fig. S1) and less frequent at the lower central position (fig. S1) in the *Varroa*-infested colonies compared to the *Varroa*-free colonies (LMM using day, time slot of recording, and hive code as random factors, Tukey post hoc test with FDR correction; *z* = 4.201, *P* < 0.001; *z* = 3.020, *P* = 0.002; respectively; Fig. 4). In all the other comb positions, no differences in the relative frequency of foraging dances occurred between *Varroa*-free and *Varroa*-infested colonies (LMM using day, time slot of recording, and hive code as random factors, Tukey post hoc test with FDR correction; *P* > 0.05; Fig. 4). Comb substrate type (cell level) was also a strong predictor of the relative frequency of foraging dances occurring in *Varroa*-free and *Varroa*-infested colonies (interaction effect between experimental group and comb substrate type; LMM using day, time slot of recording, and hive code as random factors; χ^2^ = 23.001, df = 5, *P* < 0.001; Fig. 5). In particular, confirming prediction 1c, the relative frequency of foraging dances on capped brood cells was much lower in the *Varroa*-infested colonies compared to the *Varroa*-free colonies (LMM using day, time slot of recording, and hive code as random factors, Tukey post hoc test with FDR correction; *z* = 3.219, *P* = 0.0012; Fig. 5). In addition, dances on pollen provisions (nonbrood cells) were much more frequent in the *Varroa*-infested colonies than in the *Varroa*-free colonies (LMM using day, time slot of recording, and hive code as random factors, Tukey post hoc test with FDR correction; *z* = 2.962, *P* = 0.003; Fig. 5).

#### Prediction 2

To test prediction 2 (Fig. 1), i.e., spatial shift of allogrooming behavior toward the core of the colony under parasite pressure, we verified whether allogrooming was performed mostly on central frames rather than on lateral ones (prediction 2a), on the central part of the comb rather than near the entrance of the hive (prediction 2b; fig. S1) and/or on uncapped brood cells rather than on food cells (prediction 2c; fig. S1) in *Varroa*-infested compared with *Varroa*-free hives. In the same videos used for foraging dances (13 hours and 30 min per group), we detected a total of 67 and 100 allogrooming events in *Varroa*-free and *Varroa*-infested colonies, respectively. The observed frequency of allogrooming did not differ between the two experimental groups (*Varroa*-free versus *Varroa*-infested) (GLMM using day, time slot of recording, and hive code as random factors; χ^2^ = 2.828, df = 1, *P* = 0.092; fig. S3). The mean number of groomers per event of allogrooming, which was approximately 1, did not differ significantly between the two groups either (GLMM using day, time slot of recording, and hive code as random factors; χ^2^ = 0.592, df = 1, *P* = 0.597).

**Fig. 6.**
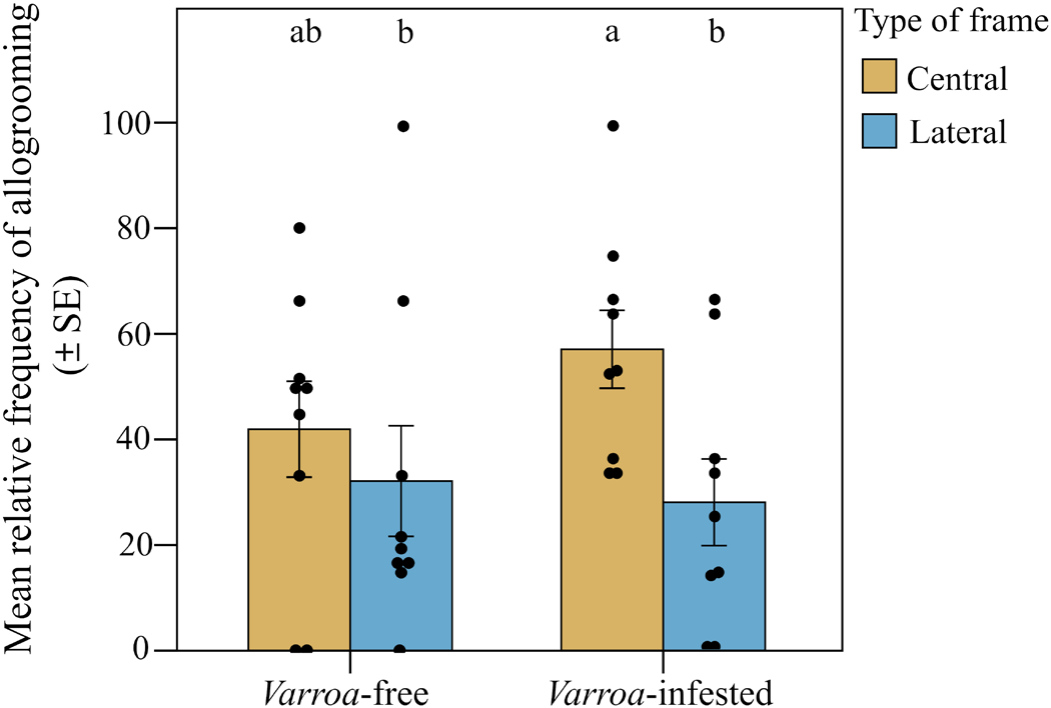
Spatial shift in allogrooming behavior. Relative frequency (means ± SE) of allogrooming events per hive and observation day detected inside the nest in *Varroa*-free and *Varroa-*infested colonies depending on the type of frame (central or lateral). Bars with different letters are significantly different (*P* < 0.05).

**Fig. 7.**
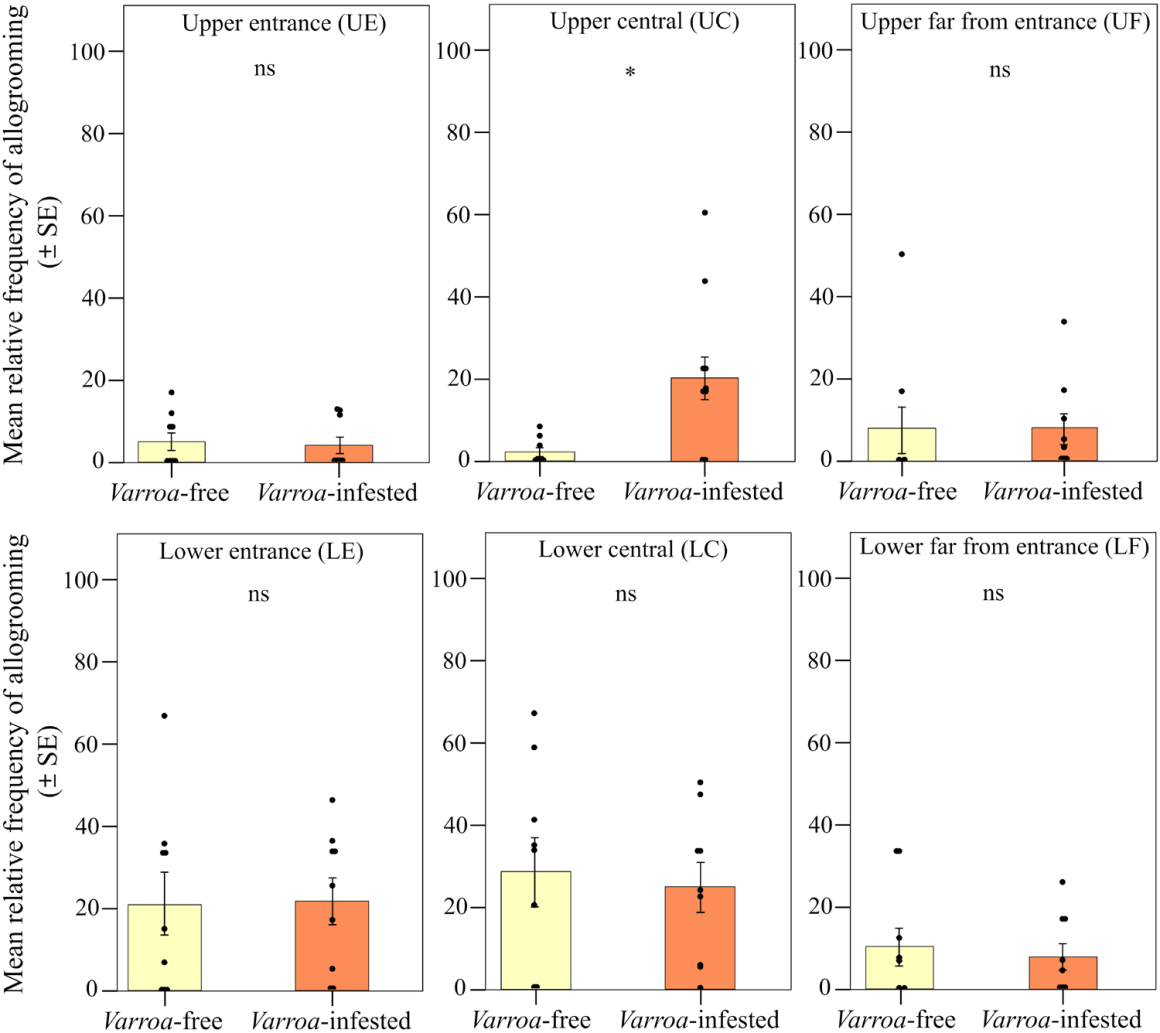
Spatial shift in allogrooming behavior. Relative frequency (mean ± SE) of allogrooming events per hive and observation day detected inside the nest in *Varroa*-free and *Varroa-*infested colonies depending on the position in the frame in relation to the hive entrance [lower and upper position near the entrance (LE and UE), lower and upper central position (LC and UC), lower and upper position far from the entrance (LF and UF)]. ns, *P* > 0.05; and *P < 0.05.

**Fig. 8.**
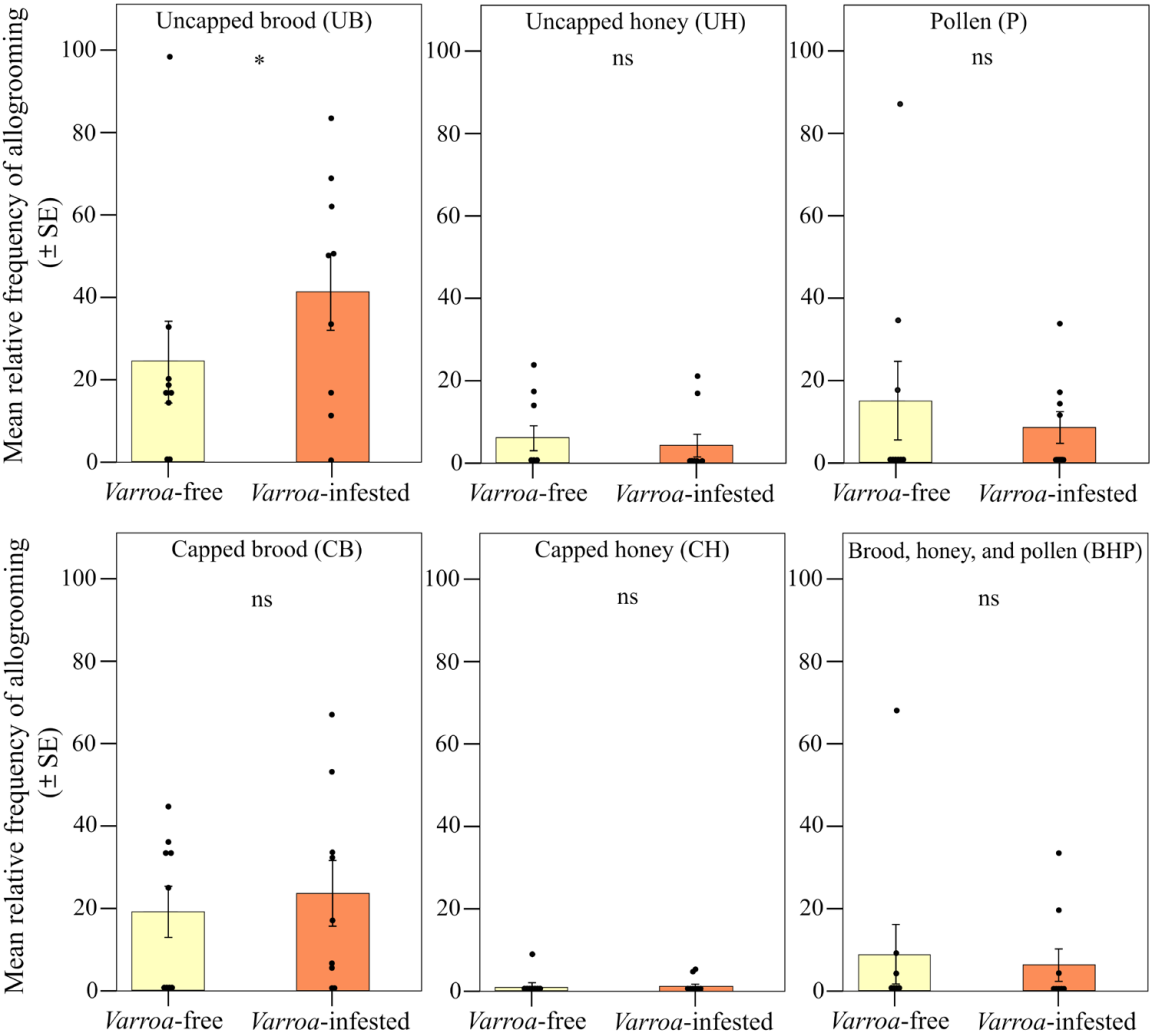
Spatial shift in allogrooming behavior. Relative frequency (mean ± SE) of allogrooming events per hive and observation day detected inside the nest in *Varroa*-free and *Varroa-*infested colonies depending on the type of substrate [uncapped and capped brood (UB and CB); uncapped and capped honey (UH and CH); pollen (P); and a mix between brood, honey, and pollen (BHP)]. ns, *P* > 0.05; and *P < 0.05.

We found strong support for our prediction 2 (Fig. 2B). Although the relative frequency of observed allogrooming events was not significantly different between *Varroa*-free and *Varroa*-infested colonies when comparing the two types of frame [interaction effect between experimental group (*Varroa*-infested and *Varroa*-free) and type of frame; LMM using day, time slot of recording, and hive code as random factors; χ^2^ = 1.407, df = 1, *P* > 0.05; Fig. 6], we found a significantly higher relative frequency of allogrooming events in the central frame compared to the lateral ones in infested colonies (prediction 2a; LMM using day, time slot of recording, and hive code as random factors, Tukey post hoc test with FDR correction; *z* = 2.536, *P* = 0.011; Fig. 6) and no differences between central and lateral frames in the uninfested group (LMM using day, time slot of recording, and hive code as random factors, Tukey post hoc test with FDR correction; *z* = 0.858, *P* = 0.390; Fig. 3A). A shift in allogrooming toward the colony core in infested colonies was also supported by the spatial analysis at the comb level (prediction 2b). Within each experimental group, allogrooming varied significantly according to position in the comb in relation to the hive entrance (LMM using day, time slot of recording, and hive code as random factors; χ^2^ = 18.813, df = 5, *P* = 0.002; Fig. 7). When comparing the *Varroa*-free and *Varroa*-infested colonies, a significantly higher relative frequency of allogrooming events occurred in the upper central position of the *Varroa*-infested colonies (LMM using day, time slot of recording, and hive code as random factors, Tukey post hoc test with FDR correction; *z* = 7.417, *P* = 0.016; Fig. 4B). For all other comb positions, no differences in relative frequency of allogrooming events were detected between the two experimental groups (LMM using day, time slot of recording, and hive code as random factors, Tukey post hoc test with FDR correction; *P* > 0.05; Fig. 7). Last, confirming prediction 2c, we observed a higher relative frequency of allogrooming events on the uncapped brood cells in infested colonies than in uninfested colonies (LMM using day, time slot of recording, and hive code as random factors, Tukey post hoc test with FDR correction; *z* = 2.345, *P* = 0.019; Fig. 8). In all other types of substrate (honey, pollen, and mix between honey and pollen), we did not find any significant differences between *Varroa*-free and *Varroa*-infested colonies (LMM using day, time slot of recording, and hive code as random factors, Tukey post hoc test with FDR correction; *P* > 0.05; Fig. 8).

### High-resolution observation of individual social behavior

#### Prediction 3

To test prediction 3 (Fig. 1), i.e., increase in individual social behaviors that normally limit the spread of the disease, such as allogrooming (prediction 3a), and a reduction in those that increase the likelihood of a spread, such as antennation (prediction 3b) and trophallaxis (prediction 3c), we conducted an experiment to compare the individual frequency of each type of behavior between caged adult bees of the *Varroa*-free group, all unparasitized (V0), and those of the *Varroa*-infested group, which included parasitized (V+) and unparasitized (V−) adult bees in equal proportion (percentage of infestation of 50%). The individual-level experiment was replicated three times, by using six independent cages (three for the infested group and three for the uninfested group), each containing 12 marked bees, in each replicate.

By checking the videos recorded on cages of *Varroa*-free and *Varroa*-infested groups (180 min of video footage per group), we detected the following: (i) 160 and 183 events of allogrooming, respectively; (ii) 1198 and 1015 events of antennation, respectively; and (iii) 889 and 654 events of trophallaxis, respectively.

**Fig. 9.**
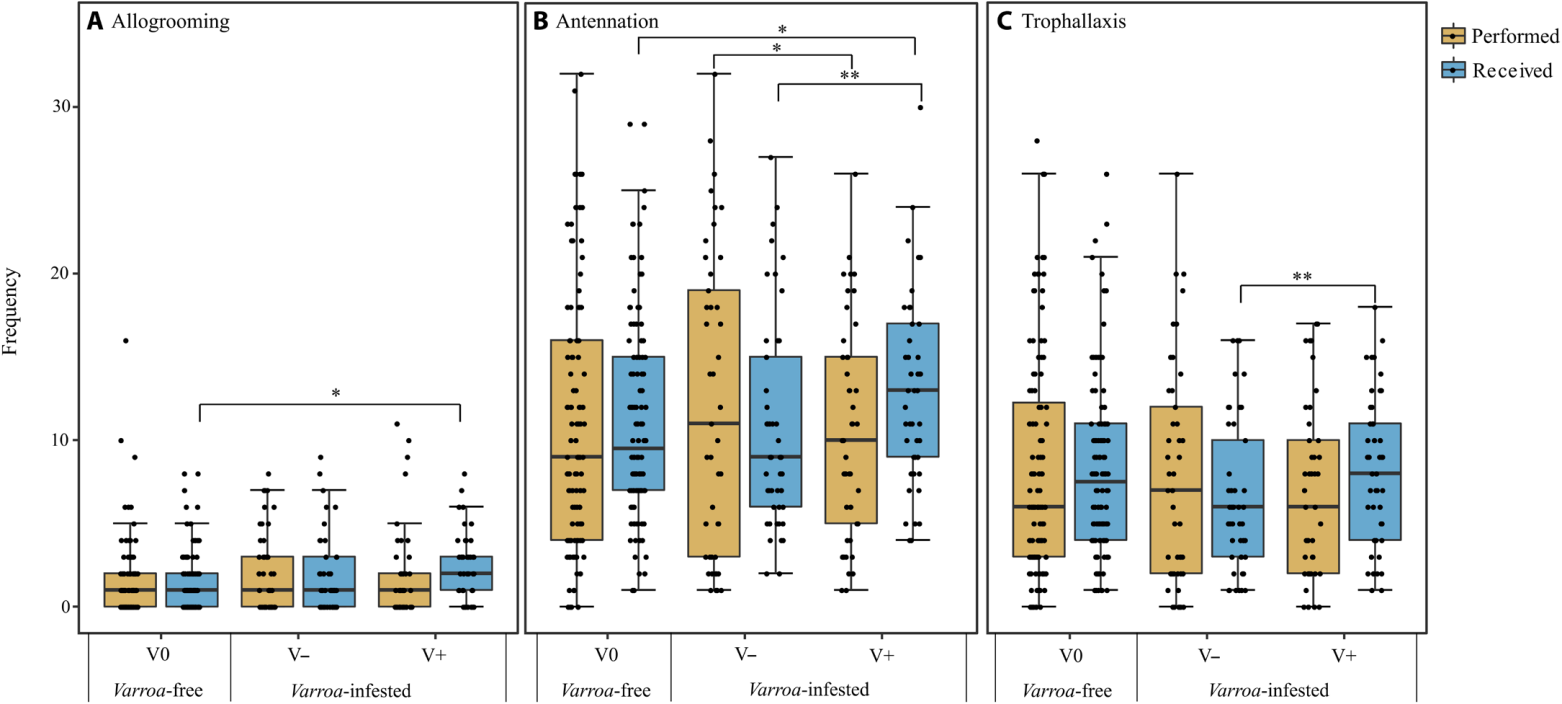
Allogrooming, antennation, and trophallaxis frequency. Individual frequency of allogrooming (**A**), antennation (**B**), and trophallaxis (**C**) events performed and received within the *Varroa*-free and *Varroa*-infested cages (box plots show median and interquartile range). V0 are unparasitized individuals of the *Varroa*-free group, whereas V− and V+ are unparasitized and parasitized individuals of the *Varroa*-infested group, respectively. *P < 0.05 and **P < 0.01.

When we looked at the individual action of the bees (focal animal sampling), the differences in the mean frequency of allogrooming between performers (givers) of the two caged groups were not significant [GLMM using cage and experiment (replicate) as random factors; χ^2^ = 2.217, df = 1, *P* = 0.136; Fig. 9A]. Differently, when receivers were considered, prediction 3a was confirmed, with significant differences in the mean frequency of allogrooming received between the *Varroa*-free and the *Varroa*-infested groups [GLMM using cage and experiment (replicate) as random factors; χ^2^ = 4.150; df = 1, *P* = 0.04; Fig. 9A]. The mean frequency of allogrooming an individual received was higher in the parasitized adult bees of the *Varroa*-infested group (V+) compared to the unparasitized adult bees of the *Varroa*-free group (V0) [GLMM using cage and experiment (replicate) as random factors, Tukey post hoc test with FDR correction; *z* = 2.393, *P* = 0.016; Fig. 9].

Prediction 3b, i.e., decrease in antennation, a behavior that increases the likelihood of parasite spread, in infested colonies was not confirmed by our observations. On the contrary, a general increase in the antennation frequency in the infested groups compared to uninfested group was recorded [GLMM using cage and experiment (replicate) as random factors; χ^2^ = 4.917, df = 1, *P* = 0.026]. This was because parasitized individuals (V+) in the *Varroa*-infested cages received more antennation compared to unparasitized ones of the *Varroa*-free cages (V0) and of the same group (V−) [GLMM using cage and experiment (replicate) as random factors, Tukey post hoc test with FDR correction; *z* = 1.968, *P* = 0.045; *z* = 2.587, *P* = 0.009, respectively; Fig. 9B]. Moreover, the mean frequency of performed or received antennation did not differ between unparasitized individuals of the *Varroa*-infested cages (V−) and unparasitized individuals of the *Varroa*-free cages (V0) [GLMM using cage and experiment (replicate) as random factors, Tukey post hoc test with FDR correction; *z* = 1.297, *P* = 0.194; Fig. 9B]. Within the *Varroa*-infested group, we noted that parasitized individuals (V+) performed less antennation compared to unparasitized ones (V−) [GLMM using cage and experiment (replicate) as random factors, Tukey post hoc test with FDR correction; *z* = 2.544, *P* = 0.011; Fig. 9B].

Prediction 3c, i.e., decrease in trophallaxis, another type of behavior that increases the likelihood of parasite spread, in infested colonies (Fig. 1) was not confirmed by our observations either. We did not find significant differences in the mean frequency of performed or received trophallaxis between *Varroa*-free and *Varroa*-infested cages [GLMM using cage and experiment (replicate) as random factors; χ^2^ = 2.622, df = 1, *P* > 0.05]. In addition, within the *Varroa*-infested group, we observed again a significantly different behavioral pattern between parasitized and unparasitized individuals, in which parasitized individuals (V+) received more trophallaxis than unparasitized individuals (V−) [GLMM using cage and experiment (replicate) as random factors, Tukey post hoc test with FDR correction; *z* = 2.763, *P* = 0.005; Fig. 9C].

#### Prediction 4

**Fig. 10.**
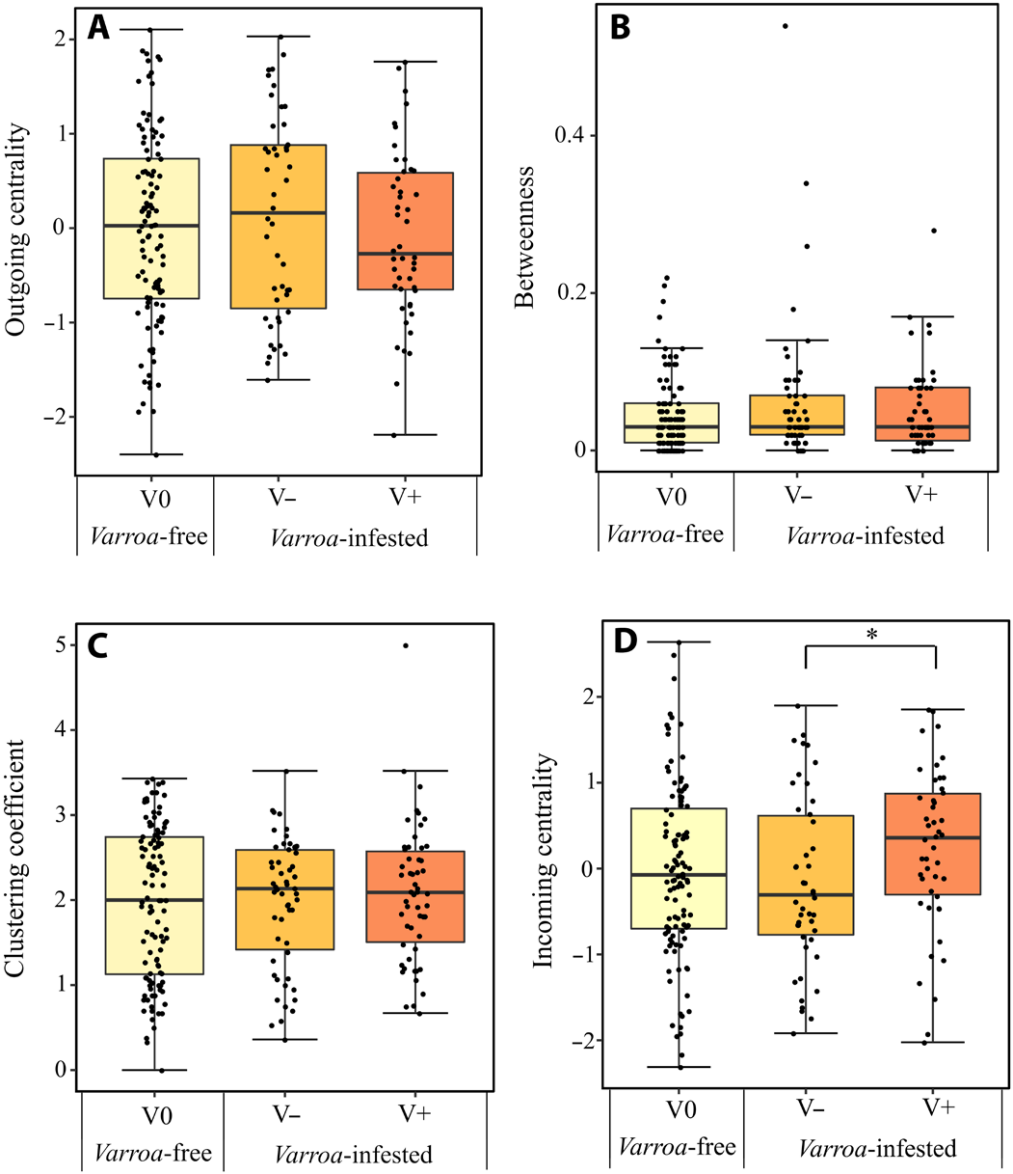
Social network parameters. Comparison of centrality measures, i.e., outgoing centrality (**A**), betweenness (**B**), clustering coefficient (**C**), and incoming centrality (**D**), among unparasitized (V0) individuals in *Varroa*-free groups and unparasitized (V−) and parasitized (V+) individuals in *Varroa*-infested groups. Box plots represent the median (line), quartiles (box), and extreme values (no greater than 1.5 times the interquartile range, whiskers). *P < 0.05.

To test prediction 4 regarding changes in social network structure in infested groups (Fig. 1), we checked whether, under parasite pressure, there was a decrease in network connectivity and node centrality at the whole network level (prediction 4a) and/or a decrease in individual centrality at the single node (bee) level (prediction 4b). Social networks of *Varroa*-free and *Varroa*-infested groups were all made by a single component and were overall tightly connected. We found no support for prediction 4a, because there was no difference in the network cohesion index between *Varroa*-free and *Varroa*-infested groups (prediction 4a; LMM using cage, experiment, and colony of origin as random factors; χ^2^ = 0.075, df = 1, *P* = 0.783; fig. S4). We found no support for prediction 4b either. Individual position in the social network did not differ among unparasitized individuals of the *Varroa*-free group (V0) and unparasitized (V−) and parasitized (V+) individuals of the *Varroa*-infested group for most of the measured metrics of centrality: outgoing centrality (LMM using cage, experiment, and colony of origin as random factors, Tukey post hoc test with FDR correction; *P* > 0.05; Fig. 10A), betweenness (LMM using cage, experiment, and colony of origin as random factors, Tukey post hoc test with FDR correction; *P* > 0.05; Fig. 10B), and clustering coefficient (LMM using cage, experiment, and colony of origin as random factors, Tukey post hoc test with FDR correction; *P* > 0.05; Fig. 10C). Only incoming centrality differed between the groups but in the opposite direction to what predicted, being higher in the V+ than in the V− group (LMM using cage, experiment, and colony of origin as random factors, Tukey post hoc test with FDR correction; *z* = 2.487; *P* = 0.03, Fig. 10D).

## DISCUSSION

Parasitic mites represent a challenge for several insect societies, and infestation from *V. destructor* is among the most serious threats for the honey bee, *A. mellifera*. The arrival and spread of the mite cause a series of marked behavioral changes within honey bee colonies. For example, it has been shown that this parasite affects brood rearing activity (*23*), accelerates temporal polyethism, resulting in early foraging activity (*37*), and reduces orientation capacity of foragers, thus increasing the drift phenomenon (*38*, *39*). To our knowledge, here, we provide evidence that *V. destructor* significantly affects the space use and the social interactions inside the hive, with infested colonies showing changes in traits associated with social immunity (space use and social interactions) at the whole colony and the individual level.

When challenged by a parasite or a pathogen, a colony might benefit from the adoption of induced organizational immunity, i.e., limiting the spread of the pathogen by increasing spatial and behavioral compartmentalization of the different cohorts of individuals (*8*, *9*). We found two lines of evidence supporting this when we monitored two behaviors likely involved in the dispersion of the parasite, i.e., foraging dances and allogrooming, in infested and uninfested colonies. First, we found that foraging dances, which can promote the entry of *Varroa* into the colony through hitchhiking on foragers’ bodies (*25*, *26*), occurred more frequently at the periphery of the hive in infested colonies compared to uninfested colonies. In the latter, foragers danced both in the central and in the lateral frames, in accordance with previous findings (*29*). When considering the position within the comb (frame level), the predominance of foragers dancing in the bottom half of the comb in both groups was probably because that area is more illuminated, as highlighted in other studies (*29*, *40*, *41*). However, in the infested colonies, foraging dances were more concentrated closest to the hive entrance, whereas in the uninfested group, the foraging dances were distributed equally in the position closest to the hive entrance and in the central portion of the comb. At the cell level (type of substrate), in the infested group, there was a relative increase in the frequency of dances performed on food cells, to the detriment of those performed on capped brood cells, whereas no changes occurred in the uncapped brood cells. This result seems to partially contradict our prediction, considering that the uncapped brood represents the substrate used by *Varroa* for reproduction. However, according to Tautz (*42*) and Tautz and Rohrseitz (*43*), when the dance is performed on open cells, such as uncapped brood cells, the vibrational signal is transmitted more distant and more effectively, thus resulting in a greater recruitment of other foragers. Therefore, our findings suggest a trade-off between the need to limit the spread of the parasite and the need to maintain an efficient communication within the colony in the infested hives. The change in space use that we observed in the presence of *Varroa* can be interpreted as a strategy to mitigate the effects of parasitosis (induced organizational immunity), as shifting foraging dances to the periphery reduces the contact between the periphery (foragers) and the core (nurses) of the colony (*8*, *9*). At the same time, an efficient communication within the colonies of the infested group is maintained by performing foraging dances on the uncapped brood as often as those observed in the uninfested group.

The second line of evidence is represented by the differences in the spatial pattern of allogrooming detected between infested and uninfested colonies. We found that allogrooming, which can reduce the spread and incidence of parasitism (*32*), occurred more frequently on central frames rather than on lateral ones, in the central areas of the comb, usually occupied by brood, and on uncapped brood in infested hives when compared to uninfested hives. In infested colonies, allogrooming would be expected to be concentrated in the core of the colony, i.e., in the region of the comb with brood, where nurses remain and where newly emerged bees carrying mites might emerge more frequently (*17*, *19*).

Two nonmutually exclusive processes might explain the spatial shift in allogrooming observed in infested colonies. First, allogrooming might be performed where mites are most likely to be found, in line with the organizational immunity theory. The mites preferentially parasitize the nurse bees to improve their fitness (*27*) and to be transported near the brood cells that they must invade to reproduce (*17*, *19*). In addition, the shift of the allogroomers toward the center of the nest in infested colonies could represent an indicator of the shift of the whole cohort of young workers toward the center of the nest, to increase the distance between the latter and the cohort of older bees (foragers) that normally occupy the outermost part of the nest.

On the basis of our results related to foraging dances and allogrooming, we hypothesize that honey bee colonies respond to the presence of *Varroa* by moving foragers to the periphery of the nest and the young bees (nurses) toward its center, thus increasing the distance between the two cohorts of bees compared to that detected in the absence of parasitism.

The analyses at the whole-colony level provided strong evidence for induced organizational immunity, whereas the results at the individual level, obtained on cohort individuals (1-day-old bees) in cage experiments, provided a mixed support. On one hand, the increased levels of allogrooming received by the *Varroa*-infested 1-day-old bees confirmed the predictions of the social immunity theory. On the other hand, the observed increases in antennation and trophallaxis observed in the infested groups compared to the noninfested groups did not confirm our predictions. The increased antennation received by infested individuals was probably related to the detection of cues emitted by the parasite (*35*, *44*), and their greater aptitude for receiving more trophallaxis than uninfested individuals of the same group could be a way of limiting the aggressive behavior of nestmates and increasing acceptance by other bees (*13*). These results contradict the prediction of a decreased level of interaction to reduce the spread of the mite. Moreover, the analysis of network structure revealed lack of significant organizational immunity strategy induced by the presence of the mite, as we did not find any evidence for a reduction in the social cohesion within the same cohort of individuals (whole network level) or for a reduction in the centrality of infested bees, compared to the other bees, within the social network. On the contrary, infested bees were even more central than the noninfested ones, likely because of the higher levels of allogrooming, antennation, and trophallaxis received. This lack of social distancing, with an even higher connectivity of infested individuals, contradicts the organizational immunity predictions, whereas an increase in caregiving could be a reasonable explanation from a social immune perspective, as this behavior might help to reduce the parasite load (*32*). However, we should consider that caregiving behavior, which requires physical contact, can have opposing effects: It can reduce infection levels by killing some of the mites attacking the infested bees, but it could also facilitate its spread to the caregivers (*45*). Our combined results indicate that social distancing occurs at large scale (i.e., colony level) but not at a smaller scale (within a cohort of bees), where caregiving behavior seems to prevail. This suggests that the trade-off between protection and risk of transmission due to social interactions might vary with the spatial scale and the cohort considered. Although we recognize that a lack of evidence is not a proof of absence, we could hypothesize that in groups located at the core of the colonial social network (e.g., our experimental groups of 12 newly emerged bees), ergonomic optimization might prevail on the need to reduce disease spread. At the core of the society, an excess of compartmentalization, by avoiding interactions with infected nestmates, might lead to social disruption and a dramatic loss of work force. Although our study does not provide evidence to support either of the two hypotheses (caregiving or ergonomic optimization), it shows the importance of investigating the variation in social immunity strategies and their interaction across different levels (e.g., cohort age) and contexts in insect societies (*46*–*48*).

Our study clearly shows that honey bee colonies respond to the ectoparasite *V. destructor* by modifying the use of space, thus increasing the social distance between the cohort of young and old bees. These findings are in line with the theory of induced organizational immunity described by Stroeymeyt *et al.* (*9*) in *L. niger* infected with the fungal pathogen *M. brunneum* and by Geffre *et al.* (*13*) in honey bees infected with Israeli acute paralysis virus. In honey bees, this dynamic is also observed at higher levels of biological organization. It was found that the horizontal transmission of *V. destructor* increases significantly as the intercolonial distance decreases (*49*, *50*). Therefore, it can be hypothesized that the high distances observed between bee colonies in natural conditions (*51*), which are much greater than those used in modern beekeeping, derive from an evolutionary path induced, at least partially, by the need to limit the intercolonial spread of diseases and to guarantee a more rational exploitation of food resources.

Social distancing as a behavioral response to disease is certainly costly for all social animals, as humanity is also experiencing during the current COVID-19 (coronavirus disease 2019) pandemic (*52*, *53*), but the widespread use of this strategy in nature suggests that the benefits may outweigh the costs (*54*, *55*). The ability shown by social insects to modulate their social structure in relation to the risk of disease transmission allows individuals to maximize the benefits of social interactions whenever possible and minimize the specific risk of infectious diseases (*54*, *55*). In addition, to minimize social costs, specific changes in the social structure can be favored (e.g., in space, frequencies, and network property) while maintaining the interaction within the group (*54*).

In conclusion, our findings on social immunity in *A. mellifera* suggest that the *V. destructor* mite triggers a behavioral and spatial variation in honey bees in response to the spread of infestation. This behavioral plasticity likely helps to find a balance between exchanging information, which is indispensable in social animals such as honey bees, and fighting the spread of diseases and parasites in the hive.

## MATERIALS AND METHODS

### Experimental apiary

The study was performed in an apiary of the experimental farm of the Department of Agricultural Sciences, University of Sassari, located in Ottava in the northwest of the island of Sardinia (40°46′23″N, 8°29′34″E) (Italy), from May 2019 to November 2019. The apiary consisted of 18 *A. mellifera ligustica* colonies: 6 colonies were maintained in observation hives (which had two glass windows, one on the right side and one on the left side) for the surveys made inside the nest, and 12 colonies were maintained in Dadant-Blatt standard hives as a source of worker bees and mites for the laboratory experiments. The Dadant-Blatt hives and the observation hives contained 10 combs each and had a nest entrance featured by a different color pattern to reduce drifting (*56*). During the experimental period, each colony was monitored every 15 days to assess the presence of the queen, brood, and food provisions. Before each experiment, the colony infestation level was monitored following standard procedure (*57*). In addition, the colony strength was assessed by estimating the total sealed brood extension and the amount of adult bees in the hive (*58*). For this purpose, one-sixth of a Dadant-Blatt frame (1880 mm^2^) was used as a unit of measure, and the number of capped cells and adult bees was obtained by multiplying the number of sixth of each matrix for 780 and 254, respectively (*58*). Nine *Varroa*-free hives were obtained by treating half of the colonies of the apiary with trickled oxalic acid every week, for three consecutive weeks, starting from 2 months before the start of the observations. In the other nine colonies, only the first treatment with trickled oxalic acid was applied, 2 months before the beginning of the observations, and then the *Varroa* infestation level grew naturally. The colony strength was balanced in both experimental groups by removing brood frames from the strongest colonies.

### Whole-colony behavioral observations

The observations on foraging dances and allogrooming were performed according to the “all occurrences sampling method” (*59*). Videos were made at the same time on *Varroa*-free (three colonies, average ± SE, infestation level 0.11 ± 0.11%) and *Varroa*-infested groups (three colonies, average ± SE, infestation level 6.2 ± 0.34%) by using four high definition (HD) cameras (Canon LEGRIA HF R506, Tokyo, Japan), two for each of the two observations hives being compared simultaneously (*60*). In three consecutive days, each colony was recorded during a 15-min session, three times a day (morning, afternoon, and evening), between 10:30 a.m. and 6:30 p.m. by placing two video cameras ~30 cm far from each glass window (on the right and left sides). A total of 27 hours of video recording was captured following a random pattern among the six hives, each one observed for the same duration (4.5 hours). The observations were made simultaneously on two types of comb selected 12 hours before starting the experiment: One was taken from the central part of the nest and presented capped and uncapped brood on at least 60% of its surface area (central frame), whereas the second was taken from the sides of the hive and had uncapped and capped honey and pollen on at least 60% of its surface (lateral frame). Both combs presented all types of substrates on their surface: capped and uncapped brood, and capped and uncapped honey and pollen. One hour before starting the video recording, the two selected combs were placed immediately behind the glass windows of each hive. Moving the central honeycombs to a lateral position for the time necessary to do video shooting does not alter their status of “central honeycombs,” as the foragers mark the positions of the honeycombs where they perform the dances after the first foraging flights and then return to the same positions to repeat them during the rest of the day (*29*, *41*, *42*). The two types of combs were observed alternately on the right and left side of the hive to avoid any position effect. Subsequently, the videos were screened using BORIS version 7.9.15 (*61*) to determine the frequencies (i.e., number of events per unit of time) of foraging dances and allogrooming events. The latter were considered only when they occurred for at least 2 s. To determine more easily the comb position in relation to the hive entrance where the observed types of behavior took place, the operators divided each observation frame into six portions of equal area as shown in fig. S1. The types of substrate where foraging dances and allogrooming occurred were also recorded: uncapped and capped brood, and uncapped and capped honey, pollen, and a mix of brood, honey, and pollen. When videos were viewed, the operator was not aware of the level of hive infestation (blind experimental plan).

### High-resolution observation of individual social behavior

#### Honey bee and mite source

The honey bees and *V. destructor* mites used in our social network bioassays were sampled from the Dadant-Blatt hives of our experimental apiary. The infested colonies were used as a source of *Varroa* mites, whereas the uninfested ones (infestation level < 1%) were used as a source of honey bee brood. To obtain emerging bees for our experiments, honey bee brood ready to emerge was collected from three *Varroa*-free colonies and kept in an incubator under controlled environmental conditions (35°C, 70% relative humidity, dark) for 20 hours (*62*). All emerging bees were fed with sucrose solution 50% (w/v) for 2 hours, before starting the experiments, to avoid that a differentiated diet could affect social networks, as noted by Naug and Smith (*6*) and Naug (*7*). Adult female mites were sampled from bee brood cells from *Varroa*-infested colonies the same day each bioassay was set up. Honey bees or mites that showed abnormal mobility, size, or color were not included in the experiments.

#### Behavioral bioassay setup

Uninfested emerging bees, originated from three unrelated *Varroa-*free colonies, were marked by using a queen marker kit composed of colored numbered tags (2 mm Ø), a nontoxic resin-type glue, and a stick. A tag, which had a color (white, pink, and yellow) that corresponded to the colony of origin of the bee and a specific number to each single bee, was glued to the thorax of each bee (bee code). To obtain noninfested or infested bees, each marked bee was then transferred to an individual petri dish, with or without *Varroa* mite, and kept there for 2 hours. The infested bees used in our biossays were only those on which the parasite was visually detectable (V+) and preferably inserted between their abdominal segments. In each bioassay, the following experimental bee groups (treatments) were formed: *Varroa*-free (V0), without parasitized bees, and *Varroa*-infested, with 50% parasitized bees, groups. Each treatment consisted of three independent metal cages with 12 bees each (12 bees × 3 replicates = 36 bees per treatment per essay). To prevent any genotypic effect, each group of 12 honey bees was composed of four bees coming from each of the three colonies. To obtain 50% of infestation level in the *Varroa*-infested group, six infested bees (V+) (two bees per colony) and six uninfested bees (V−) (two bees per colony) were placed in each cage of this treatment. Each bioassay was replicated three times for a total of 108 bees per treatment. Each metal cage (10 cm by 10 cm by 5 cm) had a sheet of bee wax comb (9 cm by 9 cm) covering its largest inner side and was closed with a glass window (10 cm by 10 cm). The bee code (tag color and specific number) of each bee provided the following information: colony of origin, experimental group, and, within the *Varroa*-infested group, bee with or without mite.

#### Behavioral observations in cages

Each group behavior was tracked by using two HD cameras (Canon LEGRIA HF R506, Tokyo, Japan) (one camera per treatment cage), for 20 min per cage. Video recording activities were conducted simultaneously in one cage of each experimental group (*Varroa*-free or *Varroa*-infested). The frequency of the following types of social behavior was registered using “focal animal sampling,” i.e., for every single bee present in the cages (*59*): antennation, trophallaxis, and allogrooming. To reconstruct the social network among individuals within the same experimental group (*Varroa*-free or *Varroa*-infested) and to compare the structure of the social network in relation to parasite exposure, the actor and the recipient of each social interaction observed were determined, for all types of social interaction (behavior). During the behavioral observations, the parasite transfer from one individual to another was also noted. In addition, the presence or absence of *Varroa* on each bee was double-checked before and after the focal sampling of 20 min. All video recording tracks were viewed in slow motion, using VLC Media Player version 3.0.11.

### Statistical analysis

#### Whole-colony behavioral observations, predictions 1 and 2

To test for differences in the frequency of foraging dances and allogrooming between *Varroa*-free and *Varroa*-infested hives, we used a GLMM with a negative binomial error structure, using day, time slot of recording, and hive code as random factors. Frequency of foraging dances and allogrooming were used as response variables and experimental group (*Varroa*-free and *Varroa*-infested) was used as a fixed factor. For the foraging dance model, besides experimental group, type of dance (round and waggle) and the interaction between experimental group and type of dance were used as fixed factors. In addition, to test whether *Varroa*-free and *Varroa*-infested hives differed in their preference to perform foraging dances and allogrooming in relation to frame position (central or lateral), position in relation to hive entrance, and substrate type, we used LMMs, with day, time slot of recording, and hive code as random factors. Relative frequency of foraging dances and allogrooming [i.e., frequency of events of a particular behavior in relation to frame position, position in the comb, or substrate type, divided by the total frequency of all events observed for the same behavior in the experimental group (infested or uninfested), multiplied by 100] were used as response variables, and experimental group (*Varroa*-free and *Varroa*-infested), spatial position, and their interaction were used as fixed factors. Furthermore, Tukey post hoc tests, adjusted for multiple comparisons using the FDR, were used to explore differences regarding the spatial position in which bees performed foraging dances and allogrooming behavior in the *Varroa*-free and *Varroa*-infested hives. In addition, we used a GLMM with a negative binomial error structure to compare the number of groomers (performers and receivers) between the *Varroa*-free and the *Varroa*-infested group, using day, time slot, and hive code as random factors. Number of groomers was used as response variable, and experimental group (*Varroa*-free and *Varroa*-infested) was used as a fixed factor.

#### High-resolution observation of individual social behavior, predictions 3 and 4

We used GLMMs with a negative binomial error structure, with cage and experiment (replicate) as random factors for the following purposes: (i) test for differences in allogrooming, antennation, and trophallaxis between individuals in *Varroa-*free and *Varroa*-infested cages. Frequencies of allogrooming, antennation, and trophallaxis were used as response variables and experimental group (*Varroa-*free and *Varroa*-infested) was used as a fixed factor; and (ii) test for differences in allogrooming, antennation, and trophallaxis behavior between individuals from the *Varroa*-free group and individuals with or without mite on their bodies from the *Varroa*-infested group. Frequencies of allogrooming, antennation, and trophallaxis were used as response variables, and category of individuals [individuals from the *Varroa*-free group (V0) and individuals with (V+) or without mite (V−) on their bodies from the *Varroa*-infested group] was used as a fixed factor. Furthermore, Tukey post hoc tests, adjusted for multiple comparisons using the FDR, were used to explore differences in allogrooming, antennation, and trophallaxis behavior between V0, V+, and V− individuals. We used separate models for performers and receivers. All mixed model analyses were performed using the R package lme4 (*63*).

All GLMM and LMM model assumptions were checked visually using residual plots (i.e., density of residuals, quantile-quantile plot, and fitted values versus the standardized residuals) generated using the mcp.fnc function within the R package LMERConvenienceFunctions (*64*) and Cook’s distance plots generated using the R package influence. ME (*65*). The model assumptions were satisfied (residuals normally distributed, homogeneity of variance, and no outliers). Tukey post hoc tests adjusted for multiple comparisons with the Benjamin-Hochberg method, to control for the FDR, were performed using the R package multcomp (*66*). All analyses were performed using R version 4.0.2 (*67*).

To test whether *Varroa* infestation induced changes in the social network of bees, we compared noninfested (*Varroa*-free) and infested (*Varroa*-infested) bee groups at two levels: (i) the cohesion of the overall social network and (ii) the individual position of each bee within its social network. We built a directed and weighted network model for each group (*N* = 18), in which nodes represented individual bees, and links between nodes represented social interactions between any two given nodes. Direction of the link went from the actor to the receiver of a behavior; e.g., for trophallaxis, it went from the donor to the receiver of the food droplet. The weight of each link was the sum of the number of interactions occurring between the two given nodes. As we were interested in the overall network of social interactions and given that some behaviors were not frequent enough to allow for network computation (e.g., allogrooming), some behaviors were highly positively correlated (antennation and trophallaxis; Pearson’s *r* = 0.913, *P* < 0.001), and individual analysis of each single behavior was performed (see above), we pooled together antennation, trophallaxis, and allogrooming for each pair of nodes. There are several measures that assess the overall cohesion of a given network. To obtain a comprehensive characterization of the structure and connectivity of the network, we computed the following metrics: (i) network density, determined as the total of all link values divided by the number of possible links; (ii) weighted clustering coefficient, a measure of the degree to which nodes in a graph tend to cluster together, calculated as the weighted mean of the clustering coefficient of all the actors, each one weighted by its degree; (iii) average weighted degree, calculated as the average of the sum of weights of the edges of nodes; (iv) *K*-core index, a measure of the robustness of a community under degeneracy, represented by the maximal subgraph in which each vertex has at least degree *k*; and (v) Wiener index, i.e., the sum of all the shortest paths between a given node and all other related nodes in the graph. In addition, at the level of dyads (subnetwork motifs), we computed triplet transitivity and arc reciprocity. The first is the number of triples that are transitive divided by the number of triplets that have the potential to be transitive by the addition of a single edge. Arc reciprocity measures the proportion of arcs that are reciprocated, i.e., if node *x* sends a tie to *y*, *y* also sends a tie to *x*.

Network cohesion measures are often positively correlated among them, and as this was our case, we applied a principal components analysis (PCA) to avoid collinearity problems. Kaiser-Meyer-Olkin (KMO) and Bartlett’s test indicated that there is scope for a PCA (KMO = 0.841, Bartlett test for sphericity *P* < 0.001). PCA produced a single principal component of eigenvalues greater than 1, which explained 85% of the total variance. Then, the resulting PC1 (which we called network cohesion index) represented all the computed network measures. Basically, the higher the network cohesion index, the more connected and tighter was the network. We then assessed the influence of treatment (infested versus not infested, *N* = 9 versus 9) using an LMM with network cohesion index as a response variable, treatment as a fixed factor, and experiment (replicate) as a random effect.

To test whether *Varroa* infestation induced changes in the individual position within the social network, we compared the individual network position (how much an individual was central in its social network) of bees of three categories: bees from *Varroa*-free groups (V0), bees without *Varroa* from infested groups (V−), and bees with *Varroa* from infested groups (V+). For each individual, we computed four standard node centrality measures (normalized within network): weighted node degree, i.e., the number of direct links that a focal individual had with other individuals, weighted by the strength of each links (number of interactions); closeness centrality, i.e., the average distance, measured as the number of edges, from a given starting node to all other nodes in the network; beta centrality, which captures the notion that a degree extends beyond just the first-order layer of connections that a focal node has by considering the centrality of the vertices to which the focal vertex is connected to; and betweenness, which measures how often a node appears on shortest paths between nodes in the network and clustering coefficient, which measures the density of a focal node neighborhood, i.e., the degree to which an individual’s immediate neighbors are connected.

We computed incoming (i.e., received) and outgoing (i.e., performed) measures for the three directional network metrics (weighted node degree, closeness centrality, and beta centrality), separately. For example, outgoing weighted degree considers only the interactions started at a specific node and directed toward another node (e.g., by the actor of an antennation), whereas incoming closeness considers only the incoming ties (e.g., by the recipient of antennation). Centrality measures of individual nodes are often positively correlated in social networks (*68*, *69*). For this reason, we applied a PCA (Varimax rotation) to the three directional network measures (weighted node degree, closeness centrality, and beta centrality) to reduce the number of variables into a smaller number of uncorrelated principal components, thus extracting principal components with eigenvalues greater than 1, and then using these as social network metrics in our study together with betweenness and clustering coefficient. KMO and Bartlett’s test indicate that there is scope here for a PCA (KMO = 0.669) and Bartlett test for sphericity (*P* < 0.001). PCA produced two principal components of eigenvalues greater than 1, which together explained 82.24% of the total variance. The resulting PC1 (which we called outgoing centrality index) was represented by weighted outdegree, outgoing beta centrality, and outgoing closeness. The resulting PC2 (which we called incoming centrality index) was represented by weighted indegree, incoming beta centrality, and incoming closeness. In a few cases, *Varroa* mites moved between individuals during the experiment, and we removed from the analysis the individuals involved (*N* = 16 of the 216 individuals in total).

We assessed the importance of category, namely, unparasitized individuals of the *Varroa*-free group (V0), unparasitized individuals of the *Varroa*-infested group (V−), and parasitized individuals of the *Varroa*-infested group (V+) on individual centrality in the interaction social network by running an LMM for each centrality measure: outgoing centrality PC1, incoming centrality PC2, betweenness, and clustering coefficient. We used each centrality measure as a dependent variable; category as a fixed factor; and cage, experiment, and colony of origin as random factors. Tukey post hoc tests adjusted for multiple comparisons using the FDR were used to explore differences between V0, V+, and V− individuals. All social network metrics were computed using UCINET v6.665 (*70*). Statistical analyses were performed using R statistical software.

## Notes

View/request a protocol for this paper from *Bio-protocol*.
