# Supplementary material for: Honey bees increase social distancing when facing the ectoparasite Varroa destructor
Source: Sci Adv. 2021 Oct 29;7(44):eabj1398. doi: 10.1126/sciadv.abj1398 (PMC8555907; doi:10.1126/sciadv.abj1398)
Supplement: Supplementary file 1 — Figs. S1 to S4 Table S1 Legend for data file S1 [file sciadv.abj1398_sm.pdf]

Science Advances
AAAS

Supplementary Materials for

Honey bees increase social distancing when facing the ectoparasite *Varroa destructor*

Michelina Pusceddu*, Alessandro Cini, Simona Alberti, Emanuele Salaris, Panagiotis Theodorou, Ignazio Floris, Alberto Satta*

*Corresponding author. Email: mpusceddu@uniss.it (M.P.); albsatta@uniss.it (A.S.)

Published 29 October 2021, *Sci. Adv.* **7**, eabj1398 (2021)
DOI: 10.1126/sciadv.abj1398

The PDF file includes:

Figs. S1 to S4
Table S1
Legend for data file S1

Other Supplementary Material for this manuscript includes the following:

Data file S1

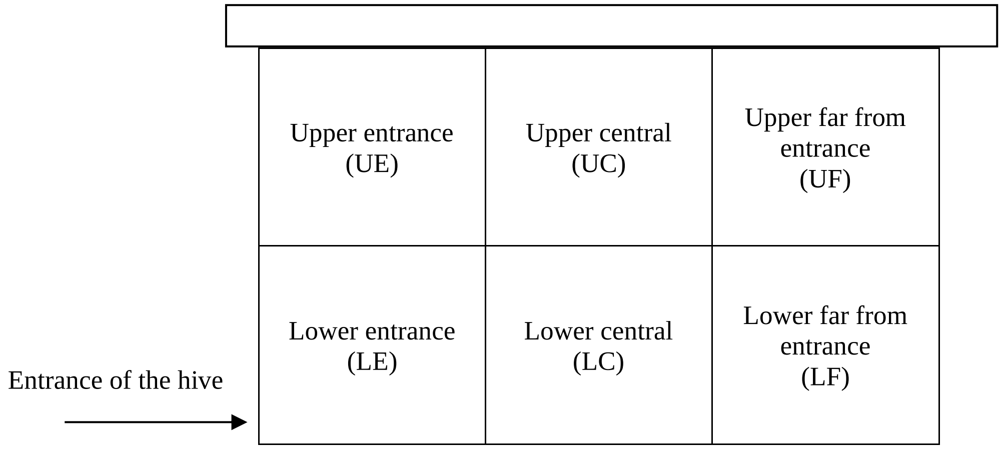

Supplementary Figure S1. **Schematic comb representation.** Suddivision of the comb used during data detection to determine the position in relation to the hive entrance where the observed types of behavior took place.

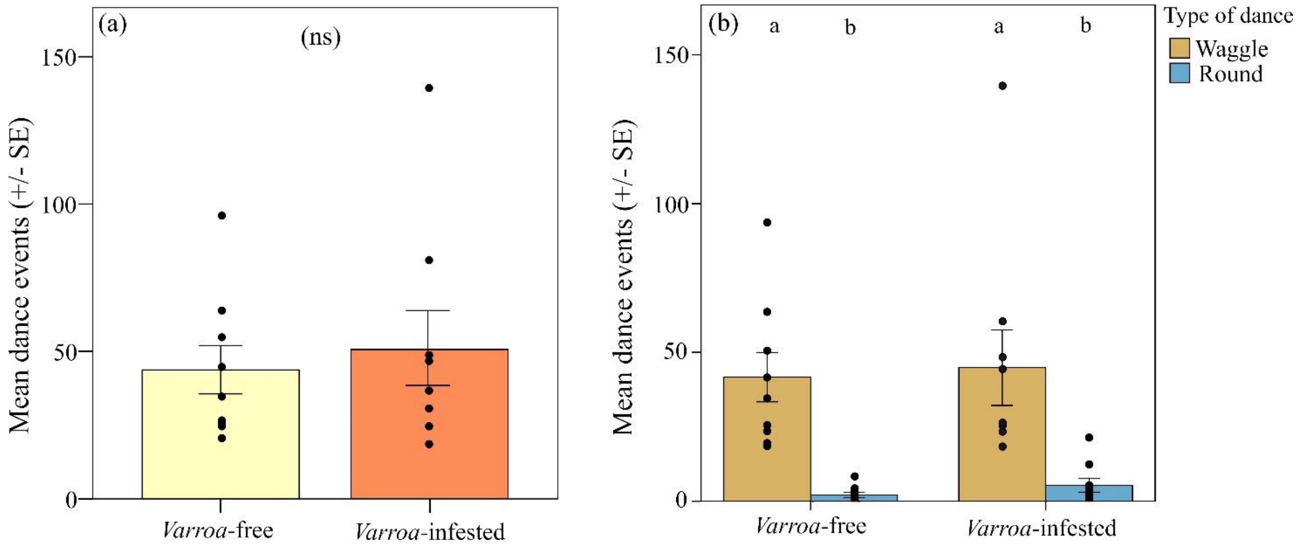

Supplementary Figure S2. **Foraging dance behavior.** Number (mean ± standard error, SE) of dance events per hive and observation day, total (a) and divided by type (waggle and round) (b), detected in *Varroa*-free and *Varroa*-infested colonies (time interval of video observation of 13 h 30 min per group). The abbreviation “ns” corresponds to not significant statistical differences at $p > 0.05$. Different letters (a, b) correspond to significant statistical differences at $p < 0.05$.

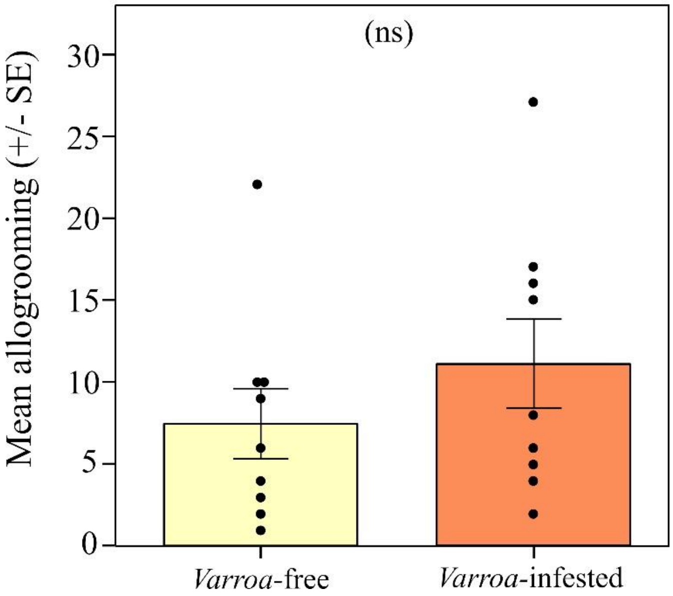

Supplementary Figure S3. **Allogrooming behavior.** Number (mean ± standard error, SE) of allogrooming events per hive and observation day detected in *Varroa*-free and *Varroa*-infested colonies inside the nest. Each hive was recorded for 45 minutes per day. The abbreviation "ns" corresponds to not significant statistical differences at $p > 0.05$.

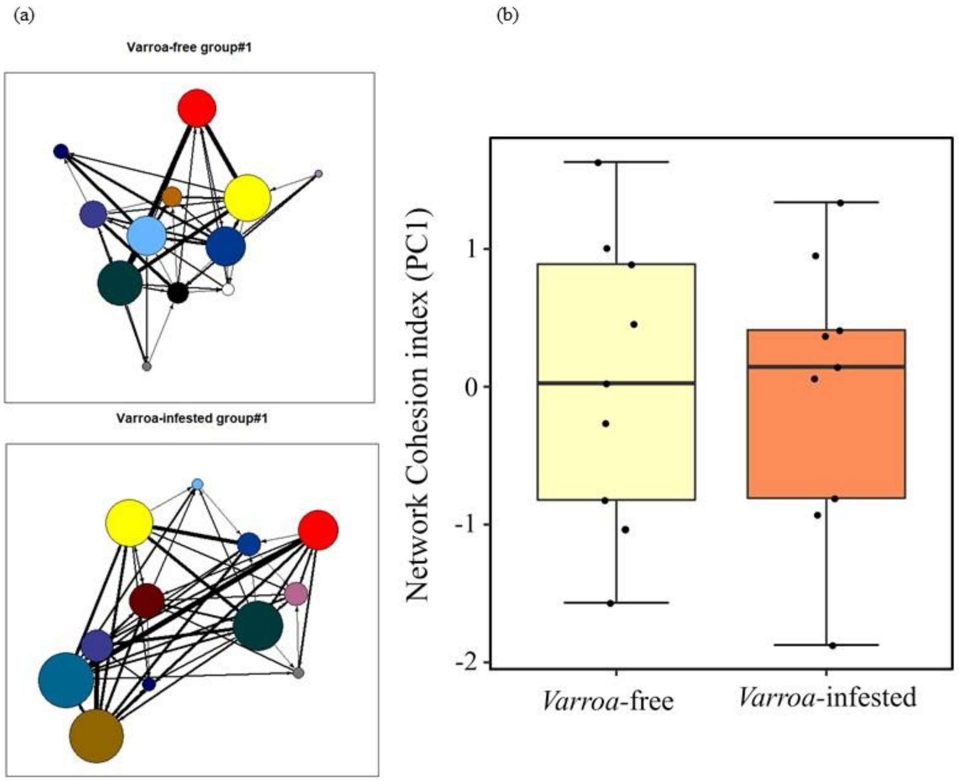

Supplementary Figure S4. **Social network structure.** The overall social network is not different between *Varroa*-free and *Varroa*-infested groups. a) An example of the social network of a *Varroa*-free and a *Varroa*-infested group. Nodes represent individual bees, links represent social interaction between nodes. Node size is proportional to the weighted degree, link size is proportional to link strength (sum of all interactions). Networks visualized according to the Kamada-Kawai force-directed algorithm. Color of nodes represent the colony of origin; b) Comparison of the Network cohesion index (see Materials and Methods) between the *Varroa*-free (N = 9) and the *Varroa*-infested groups (N = 9). No significant difference was found.

Supplementary Table S1. **Infestation and strength in observed hives.** Adult infestation level and strength of colony (mean ± standard error) in the *Varroa*-free and *Varroa*-infested groups used in the observations inside the bee nest.

**Experimental groups**	**Colonies (N)**	**Colony strength (N)†**	**Adult bee infestation level (%)**
Varroa-free	3	22581 ± 1761 a	0.11 ± 0.11 a
Varroa-infested	3	21793 ± 3168 a	6.18 ± 0.34 b

Different letters in the same column indicate significant differences (Mann-Whitney *U* test, $p < 0.05$).
†Colony strength was calculated adding the number of sealed brood cells to the number of adult bees.

Data S1. (separate file)

Datafile: dataset_bees_social_distancing

Raw data for all experiments performed in this study
